# CPD Aligned to Competency Standards to Support Quality Practice

**DOI:** 10.3390/pharmacy5010012

**Published:** 2017-02-25

**Authors:** Rose Nash, Wendy Thompson, Ieva Stupans, Esther T. L. Lau, Jose Manuel Serrano Santos, Natalie Brown, Lisa M. Nissen, Leanne Chalmers

**Affiliations:** 1 School of Medicine, University of Tasmania, Private Bag 34, Hobart, TAS 7000, Australia; leanne.chalmers@utas.edu.au; 2 School of Clinical Sciences, Queensland University of Technology, 2 George St., Brisbane, QLD 4000, Australia; wendy.thompson@qut.edu.au (W.T.); et.lau@qut.edu.au (E.T.L.L.); manuel.serranosantos@qut.edu.au (J.M.S.S.); l.nissen@qut.edu.au (L.M.N.); 3 School of Pharmacy, University of Queensland, Brisbane, QLD 4102, Australia; 4 School of Health and Biomedical Sciences, RMIT University, P.O. Box 71, Bundoora, VIC 3083, Australia; ieva.stupans@rmit.edu.au; 5 Tasmanian Institute of Learning and Teaching, University of Tasmania, Sandy Bay, TAS 7005, Australia; natalie.brown@utas.edu.au

**Keywords:** competency, continued professional development, lifelong learning

## Abstract

As medication experts, pharmacists are key members of the patient’s healthcare team. Pharmacists must maintain their competence to practice to remain responsive to the increasingly complex healthcare sector. This paper seeks to determine how competence training for pharmacists may enhance quality in their professional development. Results of two separately administered surveys (2012 and 2013) were compared to examine the reported continued professional development (CPD) practices of Australian pharmacists. Examination of results from both studies enabled a focus on how the competency standards inform CPD practice. In the survey administered in 2012, 91% (*n* = 253/278) pharmacists reported that they knew their current registration requirements. However, in the survey administered in 2013, only 43% (*n* = 46/107) reported utilization of the National Competency Standards Framework for Pharmacists in Australia (NCS) to self-asses their practice as part of their annual re-registration requirements. Fewer, 23% (*n* = 25/107), used the NCS to plan their CPD. This may be symptomatic of poor familiarity with the NCS, uncertainty around undertaking self-directed learning as part of a structured learning plan and/or misunderstandings around what CPD should include. This is supported by thematic analysis of pharmacists’ social media comments. Initial and ongoing competence training to support meaningful CPD requires urgent attention in Australia. The competence (knowledge, skills and attributes) required to engage in meaningful CPD practice should be introduced and developed prior to entry into practice; other countries may find they are in a similar position.

## 1. Introduction

As health professionals, the public holds pharmacists accountable for maintaining their knowledge, skills and attributes (competence) to practice with each personal interaction—be it at the hospital bedside, in a community pharmacy, general practice (general practice provides person centred, continuing, comprehensive and coordinated whole-person health care to individuals and families in their communities (accessed on 13 January 2017 at http://www.racgp.org.au/becomingagp/what-is-a-gp/what-is-general-practice/)) clinic or when administering a vaccination. This accountability directly translates to patient safety. For most health professionals competence, practice and Continued Professional Development (CPD) are inseparable. Competence has many definitions and meanings in the literature [1]. The Australian Pharmacy profession defines it as follows:

‘*Competence to mean that an individual possesses the required knowledge, skills and attributes sufficient to successfully and consistently perform a specific function or task to a desired standard … Inherent to the concept of competence is the inference of assessment of performance in a given circumstance against a specified external measure.*’

[2] (pp. 4–5)

For Australian pharmacists this external measure is the National Competency Standards Framework for Pharmacists in Australia (NCS) [2] and the Professional Practice Standards [3], as shown in Table 1.

The National Safety and Quality Health Service Standards, 2012 describe competency-based training as ‘*an approach to training that places emphasis on what a person can do in the workplace as a result of training completion’* [4] (p. 8). For Australian pharmacists this ‘training’ would usually include university studies with experiential placements, a supervised internship and an individual’s ongoing CPD. In this paper the authors have intentionally separated competency-based training into two elements: skills development for lifelong learning and the overall competence of the individual to practice. Whilst it is recognized that the skills for lifelong learning are essential to the maintenance of one’s competence, this paper will be focused on the competence-based training required for meaningful lifelong learning.

As described elsewhere in the literature, lifelong learning and CPD are often considered interchangeable terms [5,6,7,8]. Jane Ryan [5] interviewed nurses, physiotherapists (physical therapists) and occupational therapists to explore their understanding of lifelong learning. Their responses were themed around continuous learning, reflection, reflective practice, personal and/or professional development. Their responses reinforce why the literature often describes lifelong learning and CPD interchangeably. In the pharmacy profession, Rouse has described CPD as a framework for lifelong learning [6]. 

Currently most health professionals, including pharmacists, self-regulate their competence to practice, thus the attributes (motivation, honesty, morals, ethical consciousness, professionalism) and skills (self-assessment, reflection, informed judgment, critical appraisal) developed pre-career will inform the quality and safety of their future practice. Professionals from other health disciplines report their motivation for participating in CPD as updating their professional knowledge, updating existing qualifications, increasing the status of the profession as a whole and demonstrating individual professional competence [5].

Traditional Continued Education (CE) delivery has been described by Konstantinides as ‘*material presented in an online or live classroom format. The learning consists of listening and reading, then applying the information to an assessment, often in the form of a multiple-choice exam.*’ He states that, in contrast, CPD ‘*asks more of the pharmacist.*’ [9] (p. 2). As discussed by Konstantinides, the American Institute of Medicine [10] identified an urgent need to reform the continuing education (CE) system in 2009, citing concern regarding poorly constructed vision, a lack of inter-professional approach to education delivery, and general concerns about regulation and evaluation of continuing education [9]. In recognition that health knowledge has an increasingly short half-life [11], ongoing learning must be targeted to support the competence of the individual in their context. Expanding scopes of practice and uncertainty around the definition and exact skills of the health professional of the future reinforce the importance and need for competence informed CPD practice. Reassuringly, the importance of lifelong learning has been highlighted by the international pharmacy community for some time, as one of the essential elements of the Eight Star Pharmacist [12].

In 2010, the Pharmacy Board of Australia (PBA) introduced the CPD framework. For Australian pharmacists CPD is currently classified into three sub-groups;

- **Group 1** (one CPD credit per hour of activity): information accessed without assessment (e.g., didactic presentations, and activities with little or no attendee interaction).
- **Group 2** (two CPD credits per hour of activity): knowledge or skills improved with assessment (e.g., activities where the participant’s acquisition of knowledge or skills can be demonstrated).
- **Group 3** (three CPD credits per hour of activity): quality or practice-improvement facilitated (e.g., activities where an assessment of existing practice (of an individual or within a pharmacy practice), and the needs and barriers to changes in this practice, is undertaken prior to the development of a particular activity. As a result, the activity addresses identified professional development needs with a reflection post-activity to evaluate practice change or outcomes resulting from the activity. Such an activity will most likely extend over a number of weeks or months [13].

Pharmacists in Australia must evidence 40 points of CPD each year (consisting of no more than 20 Group 1 points) [13]. A mandatory requirement, as outlined in the CPD standards, is that all Australian pharmacists must self-assess against the NCS to identify their individual learning needs. The Pharmacy Board of Australia describes CPD as ‘*the means by which members of the profession continue to maintain, improve and broaden their knowledge, expertise and competence, and develop the personal and professional qualities required throughout their professional lives*’ [13].

**Figure 1:**
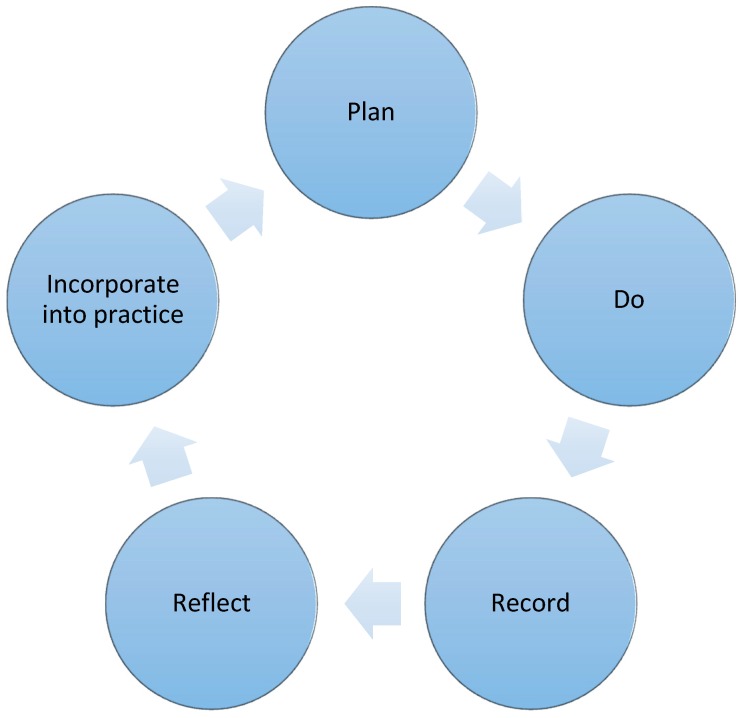
The Pharmacy Board of Australia CPD plan/record framework [15].

In December 2015, the Pharmacy Board of Australia announced the requirement that all Australian pharmacists provide evidence of a learning plan. Based on the principles of Kolb’s learning cycle [14] the Pharmacy Board of Australia CPD framework consists of five steps. **Plan**: In considering their professional role and services provided, pharmacists are to identify and document professional development opportunities. **Do**: Pharmacists then carry out a range of activities related to their scope of practice and professional development needs. **Record**: A record of CPD activities is to be made and kept for three full CPD cycles. **Reflect**: Pharmacists must consider how the activities have impacted their practice. **Incorporate**: Pharmacists then must ensure the insight and learning from CPD is actively incorporated into future practice. These steps are described in Figure 1.

Given this context, it is clear that to ensure patient safety and quality service provision and to support the advanced practice aims of the pharmacy profession, educators need to replace passive knowledge transfer approaches with self-directed learning approaches. In particular, educators must support pharmacy students, with developing skills and attributes alongside and interwoven with the requisite expert knowledge. On the whole, higher education appears to have accepted this challenge, evidenced in the move towards outcomes-focused learning rather than the traditional input-based model, which traditionally focused on an indicative curriculum [16,17,18]. In the medical profession outcomes-based education has been accepted since the 1990s [17]. The dialogue from accreditation agencies including the Tertiary Education Quality Standards Agency and professional bodies such as the Australian Pharmacy Council [19] also endorses this approach to learning. In addition, the recent emphasis on work-integrated learning [20,21], project-based assessment [22], portfolio assessment [23,24,25] and ‘authentic assessment’ strategies [26] give confidence in our ability to support graduates from all disciplines to succeed in the ‘real world’. This approach to learning and assessment can better provide pharmacy graduates with the necessary skills and attributes to survive the continual change and complexity inherent in our health system. Of relevance, this complexity is predicted to increase.

As previously highlighted by Fernandez et al. not all educators support the movement towards competency based education, and their arguments deserve mention [27]. Given the importance of these issues to the Australian Pharmacy profession, this research sought to determine:

1. How Australian pharmacists understand the CPD framework;
2. How their CPD is being guided by the NCS;
3. Whether pharmacists employ best practice strategies in their Professional Development;
4. What education models can improve the quality of CPD practice in the future.

## 2. Materials and Methods

### 2.1. Methodology

The study utilised a pragmatist frame and concurrent strategy of enquiry [28]. Consistent with this research approach, two separate surveys were administered online; each captured quantitative data around the understanding and use of the CPD framework, demonstrating multiple viewpoints from the profession. In addition, Survey 2 also captured qualitative responses, further exploring these views. Whilst anecdotal, the social media response to the Board’s announcement of CPD plans provides an interesting narrative. These findings were triangulated to explore the use and understanding of the Australian CPD requirements for greater meaning. Triangulation of ‘*data sources is a means for seeking convergence across qualitative and quantitative methods*’ [28] (p. 15).

### 2.2. Method

#### 2.2.1. Survey 1. How Is the CPD Framework Understood by Pharmacists?

WT worked in collaboration with the PBA, who reviewed the questions and advised on the content of the questionnaire. The online survey was piloted with a small group of practicing pharmacists and adjusted and amended accordingly before disseminating (September 2012 to end of October 2012). Links were made available in newsletters of the Pharmaceutical Society of Australia (PSA), the Society of Hospital Pharmacists of Australia (SHPA), and the PBA. To increase the response rate, a paper-based version was also disseminated in September 2012 to pharmacists attending two different CPD seminars in Brisbane, hosted by the SHPA and PSA, respectively. The sampling technique employed was non-probability sampling [29] and targeted Australian Registered Pharmacists. Responses were summarised using descriptive statistics. Ethics approval was granted by the University of Queensland’s Human Research Ethics Committee (2012000467). For a full list of survey questions refer to Appendix A.

#### 2.2.2. Survey 2. Current Knowledge, Use and Acceptance of the NCS by Australian Pharmacists

This online survey was open from November 2013 to June 2014 and invited all Australian students, interns, educators and registered pharmacists to participate. The sampling technique combined snowball and convenience sampling [30] and was disseminated using a combination of social media and conference presentation. As described in greater detail elsewhere [31], participants that were interviewed for a related project were also invited to distribute the survey to their networks via email. Qualitative responses were analysed using thematic analysis [32]. Quantitative responses were analysed using non-parametric techniques in SPSS V22 software (IBM: Armonk, NY, USA, 2013). Minimal risk ethics approval was obtained from the Tasmanian Social Sciences Human Research Ethics Committee (H13591). For a full list of survey questions refer to Appendix B.

#### 2.2.3. Social Media Comments Posted on Australian Pharmacist Forums

Independently, RN and IS searched commonly accessed social media forums for pharmacists for comments on CPD plans. The comments made by pharmacists on board requirements to complete a CPD plan were identified by RN and IS and combined and duplicates removed. Search terms utilised included; CPD plan, Pharmacy and Australia. The dates were intentionally restricted to August 2016 to coincide with the media releases by the PBA and the week leading up to the end of the CPD cycle. The comments were analysed independently by three authors (IS, MS, NB) using thematic analysis techniques [32]. Emergent themes were discussed for consensus and are reported in Figure 3. All comments are provided in Appendix C.

## 3. Results

### 3.1. Response Rate

#### 3.1.1. Survey 1. How Is the CPD Framework Understood by Pharmacists?

A total of 278 registered pharmacists responded to the survey representing approximately 1% of registered Australian pharmacists (*n* = 25,944). All responses were included, even though some were only partially completed [33,34].

#### 3.1.2. Survey 2. Current Knowledge, Use and Acceptance of the NCS by Australian Pharmacists

Of the 660 online survey responses, 413 were full responses and 247 were incomplete; 527 participants (who responded to five or more questions) were included. Whilst the original survey invited provisional (intern) pharmacists, educators, students and registered pharmacists, only the responses from registered pharmacists (including preceptors who are by definition registered pharmacists) will be reported on here. The results from all respondents are reported elsewhere [31]. This sample (*n* = 158) represented less than 1% of registered Australian pharmacists.

#### 3.1.3. Social Media Comments Posted on Australian Pharmacist Forums

The social media comments from a sub-section of the Australian pharmacist population (totalling 55 comments) were harvested from the most commonly accessed professional pharmacy forums (Pharmacy news, AJP.com.au). These comments were posted on four separate forums between 22 and 29 August 2016.

### 3.2. Participant Demographics

In Survey 1, respondents’ ages ranged from 20 to 65+ years, with all 10 age brackets represented and age distribution correlated to the national pharmacy census data reported by Australian Health Practitioner Regulation Agency (AHPRA) in 2011 [33]. Sixty-six percent were female, 30% were male and 4% did not disclose their gender. Fifty-six percent of respondents had been on the Australian pharmacist register (after their intern year) for more than 10 years, 16% for six to 10 years, 24% for up to five years and 4% unknown. Fifty-four percent of participants identified their primary area of practice as community pharmacy, 27% were primarily practising as hospital pharmacists, and the remaining 19% were split across a number of sectors, e.g., pharmaceutical industry, consultancy or academia. Demographics of survey respondents for Survey 2 are summarised in Table 2.

### 3.3. Survey Results

#### 3.3.1. Survey 1

The majority of respondents were accepting of the CPD framework and felt they understood the requirements. These findings have been reported in greater detail elsewhere [34]. However, when more specific questions were asked to discern their understanding of the CPD framework and its intended use, it was clear that there were gaps in the responding pharmacists’ knowledge (Table 3).

#### 3.3.2. Survey 2

**Figure 2:**
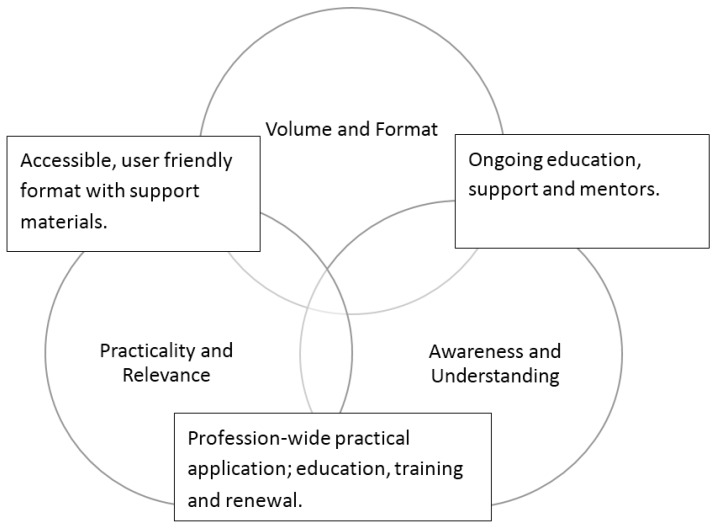
Themes derived from respondents reported barriers (circles) and enablers (rectangles) to use of NCS [31].

Overall, despite many pharmacists confirming knowledge of the NCS [31], pharmacists’ familiarity with the profession’s NCS was found to be sub-optimal (Table 4). Just over half of the responding pharmacists reported that they did not use the NCS for renewal of annual registration (57%, 61/107). The majority confirmed they did not use the NCS when planning their CPD (77%, 82/107). Their barriers and suggested solutions to use of the NCS, as reported elsewhere [31], are provided in Figure 2.

Whilst the details and sample comments from the participants are provided elsewhere [31], a summary of the themes that emerged from the thematic analysis is presented in Figure 2 below.

#### 3.3.3. Social Media Comments

**Figure 3:**
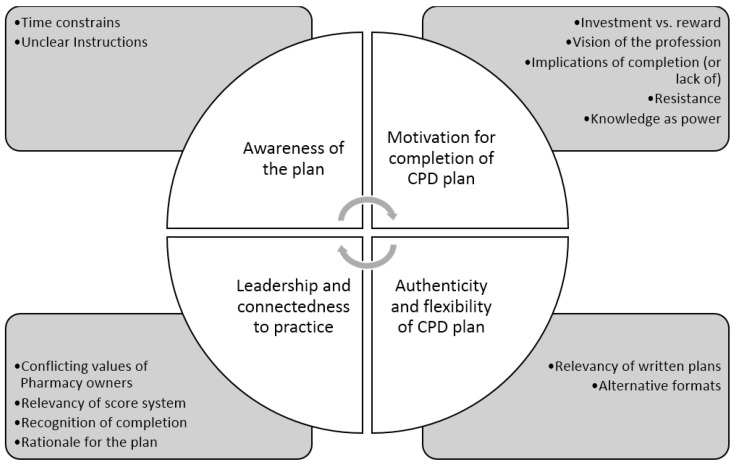
Themes derived from thematic analysis of social media comments.

There was good consensus in the thematic analysis carried out by three independent authors, which led to the development of four clear themes, presented with their sub-themes in Figure 3.

#### 3.3.4. Triangulation of All Three Sources

The major issues identified in the surveys included: “I know how to undertake self-directed learning as part of a structured learning plan” (Survey 1) with poor familiarity of the NCS and use of them to plan CPD (Survey 2). These issues align with the ‘instructions’, ‘connectedness to practice’ and ‘relevance’ sub-themes in the thematic analysis (Figure 3), which also provides additional depth around further issues. There is also some crossover in the themes from the social media comments and the qualitative aspect from Survey 2: for example, pharmacists questioning the relevance of CPD plans/NCS, concern regarding adequate awareness and understanding of each. Triangulation of the three sources highlights that respondent pharmacists want mentoring, support, education and clear instruction on the professions’ expectations around CPD plans, lifelong learning and the use of NCS in the process.

## 4. Discussion

How can competence training enhance quality in professional development? Increasingly there is a recognition that universities are now responsible for facilitating a graduate’s development of their knowledge, skills and attributes (competence). As evidence of this, the Australian Qualifications Framework [35] states that a graduate at bachelor’s level and above will be responsible and accountable for their learning needs. One essential skill desirable for all health professionals, including pharmacists, is their ability to engage in lifelong learning. This requires appropriate CPD habit formation and the development of metacognitive strategies such as self-assessment, informed judgment and reflection on action [36]. The Australian Pharmacy Council requires Australian pharmacy courses to provide evidence that their courses align with the NCS [19]. Although this is the case, this may not always be explicit to the student [37].

### 4.1. Pharmacists

Our findings highlight that prior to the introduction of the compulsory CPD plan (December 2015) the respondent pharmacists’ understanding and engagement with the CPD framework was not optimal. This sub-group of Australian pharmacists reported that they had failed to comply with the mandatory requirement set by the PBA to self-assess their practice against the NCS at their annual re-registration. They also did not appear to understand aspects of the CPD framework, as reflected in their perception on how CE (distinct pockets of learning) differed from CPD. At the time Survey 2 was administered (2013–2014), pharmacists appeared to be indicating that they were even less inclined to use the NCS to inform their CPD [34]. It is acknowledged that this finding preceded the December 2015 PBA announcement that the CPD plan/record framework would be a mandatory requirement for all Australian pharmacists. These findings highlight that some pharmacists may not appreciate the essential link between their practice, the NCS, CPD and the importance of tying all three together in meaningful CPD plans and active reflective learning cycles.

The 76% agreement that CE and CPD were equivalent in Survey 1 suggests CPD was just a number of CE events to get credits and that the pharmacists who responded did not appreciate the role of the intended learning cycle. In addition, Survey 2 suggests that the Australian pharmacists who responded were not using the NCS to assess their competency to practice despite this being mandated. The profession’s suggested barriers and enablers (Figure 2) derived from the same survey data and reported elsewhere [31] help to explain why use of the NCS in CPD planning may have been sub-optimal. These barriers were reinforced by pharmacist comments on social media (August 2016) following the release of CPD plan requirements by the PBA.

Whilst anecdotal in nature, the 2016 responses on social media (Appendix C) highlight that some Australian pharmacists are not meaningfully engaging in CPD. These comments may provide insight into the survey results. For this sub-group of pharmacists who shared their 55 comments, CPD planning is perceived as burdensome, time-intensive, inconsistent with how and what they need to learn for their practice and an insult to them as a professional. Some commented that the PBA’s requirement to develop a CPD plan is asking too much based on pharmacists’ wages and is over and above other health professionals’ CPD requirements. It is acknowledged that this sample may not represent the views of all Australian pharmacists and formal research is required to expand on this narrative further. Despite the small sample size, the results from this analysis are in alignment with those from the surveys and highlight views that are relevant to at least a part of the profession.

### 4.2. Education on NCS and Learning Cycle

These findings identified gaps in participating Australian pharmacists’ knowledge on the competent use of the CPD framework, and align with issues recognized internationally in CPD practice. In a review of CPD practice in the United Kingdom, Donyai et al. described that the understanding of fundamental aspects of the CPD process appears to remain an issue in pharmacy. Examples of barriers to conducting CPD include the inability to distinguish between CE and CPD, difficulties in assessing one’s own learning needs, and difficulty reflecting and evaluating one’s learning [38]. This indicates that an optimal CPD process will require guidance and preparation, and questions whether pharmacy curriculum is currently providing enough guidance on its use. Curricula should be equipping Australian pharmacists to have a better understanding in these areas of the NCS and CPD framework, especially as there is clear evidence that engagement in self-regulated learning can result in better learning outcomes [39,40,41]. A pilot study carried out in the United States has identified that appropriate training and support can facilitate competence in the use of a CPD approach to lifelong learning and professional development. With guidance, study participants were using specific, measurable, achievable, relevant, and time-sensitive (SMART) objectives and developing a more structured learning plan with a specific timeline and outcomes in mind. They were confident in their planning and were able to pick activities that met their objectives, rather than just selecting the most convenient activity [42]. Introduction of an e-portfolio, which provides reference to the NCS and a structured approach to document learning and development, has the potential to be important in the development of pharmacists prior to registration. This tool facilitates a platform to direct the assessment of skills required and matches and records learning to the appropriate areas of NCS competence that is being developed. This guided approach could combat the issues that have been identified in the response to the CPD plan and those previously identified in the identification, documentation and inefficiency of pharmacists’ CPD processes [38]. Providing clear instruction and increasing efficiency of the process may improve acceptance and usability.

To ensure a commitment to lifelong learning, the pharmacy curriculum must also emphasise the importance of CPD, especially as motivation to carry it out is not clear to some pharmacists (see themes identified in response to CPD plan). Assessment of their process in implementing a cycle of CPD planning could be one way of encouraging students to take the activity seriously, as it is widely recognised as a way of achieving student learning outcomes [41,43]. Formative assessment with feedback and summative assessment could provide students with the basic skills that will allow them to continue to learn and carry out CPD [41,43]. Educators should strive to emulate a motivation for learning and endeavour to share their CPD processes or skills. Additionally, as accreditation aims to produce graduates with the required knowledge and skills for internship, educators must consider whether enough guidance is being provided to our future pharmacists. It is not realistic to assume that Australian pharmacy students and even pharmacists have the requisite self-assessment skills to carry out CPD, given that international literature suggests students and registered pharmacists find self-assessment complex and challenging [44,45,46].

### 4.3. Limitations

Survey response rates were poor and likely represent more motivated individuals, resulting in selection bias. Whilst appropriate to the research questions and methodology chosen, the sampling techniques employed here (snowball, convenience and probability sampling) are likely to result in sampling bias, as are the low number of responses. The authors acknowledge the survey respondents may not represent the views of all Australian pharmacists. Similarly, the social media comments are a sub-sample and thus do not represent all Australian pharmacists. Whilst the social media forums are targeted at pharmacists, there is the potential for non-pharmacists to contribute to these forums given that they are publicly available. This research is specific to an Australian context, so potentially has limited transferability to an international audience.

### 4.4. Future Research

Future research should include longitudinal studies to explore the effect of early introduction (beginning of undergraduate studies) and meaningful use of the NCS, CPD (reflective cycles), and self-assessment on practitioner CPD practices. Investigation into whether learning plans informed by NCS correlate with pharmacist competence is required, as is a deeper understanding of what encourages competence and personal and professional development. Potentially useful to accreditation agencies is the identification of what motivates pharmacists to maintain competence and engage in lifelong learning, asking specifically if it is their job, peer pressure, patient centeredness, compliance attitudes or other motivators. Pharmacists could be surveyed to establish if the introduction of compulsory CPD plans in Australia in 2015 has improved their understanding and acceptance of CPD and led to increased use of the NCS in CPD practice. Larger-scale surveys with the pharmacy profession to further explore the themes identified from the social media analysis are required to confirm if the themes are representative of the general opinion of the profession on the implementation of CPD plans.

## 5. Conclusions

Currently some Australian pharmacists are not familiar with their NCS. Pharmacists also have limited understanding of the CPD framework. Of concern, a profession’s mandatory requirements around self-regulation of competence are not always upheld in practice. Introduction of both elements (NCS and CPD Framework) earlier, during undergraduate studies, may translate to familiarity and more meaningful use through appropriate CPD habit formation. This is one example of how competence training may enhance quality in professional development. This finding may be applicable to all pharmacy educators internationally.

## Figures and Tables

**Table 1:** National Competency Standards Framework (2010) and Professional Practice Standards (2010) for Australian Pharmacists [2,3].

National Competency Standards Framework
1. Professional and Ethical Practice
2. Communication, Collaboration and self-management
3. Leadership and Management
4. Review and supply prescribed medicines
5. Prepare pharmaceutical products
6. Deliver primary and preventative health care
7. Promote and contribute to optimal use of medicines
8. Critical analysis, research and education
**Professional Practice Standards**
1. Fundamental Pharmacy Practice
2. Managing Pharmacy Practice
3. Counselling
4. Medication Review
5. Dispensing
6. Indirect Pharmacy Services
7. Dose Administration Aids Service
8. Services to Residential Care Facilities
9. Continuity of Care through Medication Liaison Services
10. Compounding (also known as Extemporaneous Dispensing)
11. Compounding Sterile Preparations
12. Provision of Non-prescription Medicines and Therapeutic Devices
13. Health Promotion
14. Medicines Information Centres
15. Pharmacy services to Aboriginal and Torres Strait Islander Health Services
16. Screening and Risk Assessment
17. Disease State Management
18. Harm Minimisation

**Table 2:** Participant demographics for Survey 2.

	Pharmacist ^~^
Completed survey	128
Incomplete survey	56
	158
State (workplace) (*n* = 151)	
TAS	49
NSW	25
QLD	37
Other	40
Professional Organisation aligned (*n* = 158) **	
PSA	110
SHPA	26
Guild	54
Other	104
Member of Professional Organisation offers accredited CPD (*n* = 158)	
Yes	145
Area of Practice (*n* = 158) **	
Academia	3
Hospital	20
Community	125
Accredited	34
Other	18
Currently Practising (*n* = 158) ^a^	
Yes	153
Years Practice (*n* = 156)	
1–5 years	47
5–10 years	30
10–15 years	18
15–30 years	34
30 years plus	27
Hours per week paid/actual (*n* = 153)	
1–10 h	6
10–30 h	32
30–40 h	64
40 h plus	51

Changes in denominator (*n*) are due to some respondents answering some questions and not others. ^~^ Pharmacist includes pharmacists and preceptors. ** Pharmacists could select more than one category in answering some questions. ^a^ Currently practicing by Australian Health Practitioner Regulation Agency (AHPRA) definition. *States*: TAS—Tasmania, NSW—New South Wales, QLD—Queensland, Other—Northern Territory, Australian Capital Territory, Victoria, Western Australia, South Australia. *Organisation*: PSA—Pharmaceutical Society of Australia, SHPA—Society of Hospital Pharmacists of Australia, Guild—Pharmacy Guild of Australia. *Other areas of practice*: Drug & Alcohol Services, Practice Support, Administration role, Prison Service, Clinical Services, Government, Education (National Prescribing Service Facilitator), Unemployed, Rural, General Practitioner, Committee Member, Pharmaceutical Industry.

**Table 3:** Survey 1. How is the CPD framework understood by pharmacists?

Statements	% Agreement
1. I know the current CPD requirements for general registration.	91% (*n* = 253/278)
2. There has been enough guidance on CPD requirements.	77% (*n* = 215/278)
3. I know how to undertake self-directed learning as part of a structured learning plan.	57% (*n* = 158/278)
4. CPD is also known as continuing education.	76% (*n* = 210/278)

**Table 4:** Survey 2. Pharmacists’ current knowledge, use and acceptance of the NCS.

Statements	% Agreement
1. I know what the NCS are.	83% (*n* = 115/139)
2. I am not familiar (not at all/not very) with the NCS.	90% (*n* = 120/134)
3. I am familiar (familiar/very familiar/extremely familiar) with the NCS.	10% (*n* = 14/134)
4. I use the NCS for renewal of my annual registration.	43% (*n* = 46/107)
5. I use the NCS to plan my CPD.	23% (*n* = 25/107)

## Appendix A. Survey 1 Questions

Dear Pharmacist,

The Pharmacy Board now requires registered pharmacists to acquire annual Continuing Professional Development (CPD) credits for renewal of registration. This online survey will aim to investigate what registered pharmacists understand about mandatory CPD by asking how you are managing to complete your annual CPD credits.

It would be appreciated and of considerable value if you could spend approximately 10 min completing this survey. Please note that the Pharmacy Board of Australia has provided the link to the survey to you on behalf of the UQ research team and names or contact details of participants will not be disclosed to the research team.

Participation in the survey is voluntary, but completing a survey will allow entry into a prize draw, which provides an opportunity to win a $50 Myers gift voucher.

If you would like to participate, then read the attached participant information leaflet (see the link at the end of question 1).This provides a more detailed explanation of what the study involves.

Participation in the survey will contribute to understanding how pharmacists are dealing with the introduction of mandatory CPD, and the resulting data may identify if further assistance or modifications could improve the process.

Thank you for considering this request.

Note: Continuing Professional Development will be referred to as CPD.

1. Before starting this survey, please can you confirm that you have read and understood the participant information leaflet and that you consent to participate in this survey? Click here for participant information.

- Yes, I have read the participant information and I agree to participate.
- No, I do not wish to participate.

To help us put your answers into context, please can you answer the following questions before you start the survey on CPD?

2. Approximately how long have you been on the register of pharmacists in Australia (after your intern year)?

- 0–5 years
- 6–10 years
- more than 10 years

3. What is your gender?

- Male
- Female

4. Please indicate your age bracket.

- 20–24 years
- 25–29 years
- 30–34 years
- 35–39 years
- 40–44 years
- 45–49 years
- 50–54 years
- 55–59 years
- 60–64 years
- 65 years or above

5. What is your main area of practice?

- Hospital
- Community
- Academia
- Not practising
- Consultants/accredited
- Other (please specify)

*This page is just for practising Pharmacists.*

6. Approximately how many hours per week do you work as a pharmacist?

- Part-time; 15 h or less
- Part-time; more than 15 h
- Fulltime

*The questions on this page ask how you found out about mandatory CPD and investigate how well you understand the process.*

7. Please show your level of agreement with the following statements. KEY: SA = Strongly Agree, A = Agree, N = Neutral, D = Disagree, SD = Strongly Disagree.

- I know the current CPD requirements for general registration.
- There has been enough guidance to outline the CPD requirements for renewal of registration.

8. Which source has provided the most information about the CPD requirements?

- Pharmacy Board of Australia
- Pharmaceutical Society of Australia
- Society of Hospital Pharmacists
- Pharmacy Guild
- Other (please specify)

*The questions on this page will explore in more detail what you understand about the CPD process and ask which type of CPD you prefer.*

9. The Pharmacy Board has classified CPD activities into three groups and each group acquires a different number of CPD credits. Do you understand how these groups are classified?

- Yes
- No
- Unsure

10. Do these groups influence which CPD activities you will do?

- Yes
- No
- Unsure

11. Not more than 50% of the annual CPD credits required for renewal of registration can be claimed by undertaking Group 1 CPD activities. Should Group 1 activities be limited?

- Yes
- No
- Unsure

12. If you have answered yes to the previous question, please indicate what you believe the limitation should be.

- less than 50%
- more than 50%
- equal to 50%

13. For the following list of CPD activities, please give them a numerical rating between 1 and 5; with 1 indicating MOST preferred and 5 the LEAST preferred.

- Attend a lecture.
- Give a conference presentation.
- Complete an online interactive case study.
- Publish an article in a journal.
- Read a journal article.
- Complete a postgraduate education course.
- View an online lecture.
- Join the board of a local pharmacy committee.
- Read an online journal and complete the MCQ assessment.
- Attend an interactive workshop.
- Introduce a new professional service into your pharmacy.
- View an online interactive lecture.

14. Do you know how to undertake self­directed learning as part of a structured learning plan?

- Yes
- No
- Unsure

15. Are you using self-directed structured learning plans to guide your CPD activities?

- Always
- Often
- Sometimes
- Never

16. Did you use self-directed structured learning plans before national mandatory CPD was introduced?

- Always
- Often
- Sometimes
- Never

17. Are you recording CPD activities in the correct format required for audit?

- No
- Yes
- Don’t know

18. Continuing professional development can also be known as continuing education.

- No
- Yes
- Don’t know

*The last few questions on this page investigate how you acquire CPD credits.*

19. Please show your level of agreement with the following statements. KEY: SA = Strongly Agree, A = Agree, N = Neutral, D = Disagree, SD = Strongly Disagree

- Since CPD became mandatory, I have increased my number of annual CPD activities.
- Since CPD became mandatory, I have participated in a wider variety of CPD activities.
- It will be difficult for me to acquire 40 CPD credits annually.

20. Which of the following make it difficult for you to acquire CPD credits? Tick more than one box if required.

- I have no difficulties in acquiring CPD credits
- Workload
- Unsure what to do.
- Time constraints
- Access to Continuing Education events
- Cost
- Other (please specify)

21. Describe the main difficulty (if any) to obtaining your annual CPD credits.

22. Why is this challenging for you?

23. What has helped you to acquire your annual CPD credits?

24. Are you a member of a professional organisation?

- Yes
- No

25. If you answered ‘yes’ to the previous question, please state which professional organisation(s) you are a member of.

This now ends the survey on CPD. Thank you for participating.

If you would like to be entered into the prize draw then answer ‘Yes’ to the next question, otherwise answer ‘No’ to finish the survey.

26. Would you like to be entered into our prize draw? Two participants will be selected to win a $50 Coles/Myer voucher?

- Yes please
- No thanks

Please enter your e-mail address in the text box and we will contact you if you are selected in the draw.

27. Please note that your e-mail address will be used for the purpose of this draw only and it will be deleted immediately after the winners are selected.

## Appendix B. Survey 2 Questions

Demographics

1. Age:
2. Sex M/F:
3. Single Professional Organisation you most closely identify with …………………………
4. Location (Post code of workplace) (Optional):
5. ITP Provider (Optional): PSA □ Guild □ NAPE □ Other □ ………………
6. University of Study (Optional): …………………………………………………………..
7. Prior Study/Other Qualifications:
8. Registered Pharmacist: YES/NO (* If NO go to Q 11)
9. Years of Practice: 1–2 □ 2–5 □ 5–6 □ 6–10 □ 10–15 □ 15+ □
10. Currently practising: YES/NO (* If NO go the Q11)
11. Area of Practice Academia □ Hospital □ Community □ Other □
12. Hours/week (paid)………………………

Survey

13. I am familiar with the following documents/resources;

**Table:**

Standards/Framework	Strongly Agree	Agree	Unsure	Disagree	Strongly Disagree
National Competency Standards Framework for Pharmacists in Australia					
Professional Practice Profile for Initial Registration as a Pharmacist					
Australian Qualifications Framework					
Science, Vet, Health Threshold Learning Outcomes					
OLT Pharmacy Threshold Learning Outcomes					

For the remainder of the survey the Competency Standards Framework for Australian Pharmacists will be referred to as the Competency Standards.

14. Do you know what the Competency Standards are? YES/NO/UNSURE
15. Can you describe these in your own words? YES/NO/UNSURE

15a) Description ……………………………………………………………

16. Have you accessed the Competency Standards document? YES/NO/UNSURE

16a) If YES; Format used; Hard Copy □ Online □ Both □ Unsure □

16b) If YES; How often? Nil □ Once □ Twice □ Weekly □ Monthly □

17. Do you think the Competency Standards are relevant to you now? YES/NO/UNSURE
18. Do you think the Competency Standards will be relevant to you in the future? YES/NO/UNSURE
19. Do you refer to the Competency Standards to compile your CPD? YES/NO/UNSURE

19a) Can you describe any Barriers? ………………………………………………

19b) Can you think of any Enablers? ………………………………………………

20. Do you refer to the Competency Standards to chart your own progress? YES/NO/UNSURE

20a) If YES; How many times did you refer to the competency standards throughout the year? Nil □ Once □ Twice □ Weekly □ Monthly □

21. How do you use the competency standards currently?
  (a) Construct CPD Plan
  (b) Tick the box at registration
  (c) Reference
22. How do you track your CPD currently?
  (a) ePortfolio
  (b) PSA Website
  (c) Excell doc
  (d) AuspharmList
  (e) Other………
23. In your own experience as a student, do you feel the Competency Standards were introduced to students in the undergraduate or masters programs? YES/NO/UNSURE

23a) Please feel free to add a comment:

24. In your own experience as an intern, do you feel the Competency Standards informed the intern training program you participated in? YES/NO/UNSURE

24a) Please feel free to add a comment:

25. Do you think your current students are familiar with the Competency Standards? YES/NO
26. The following statements refer to various aspects of your practice. I am most interested in obtaining your candid opinions to these statements. Please choose one of five possible responses for each statement about continued competency:

**Table:**

Question	Strongly Agree	Agree	Unsure	Disagree	Strongly Disagree
I can maintain an acceptable standard of practice without attending continuing education programs.					
Continuing education such as self-study or seminars is essential for my work.					
My daily practice is all the continuing education I need.					
I would attend continuing education seminars only if they were required for re-licensure.					
Continuing education is of little importance to my practice.					
My practice would suffer if I did not attend continuing education programs.					

Adapted from [47].

## Appendix C. Social Media Comments

**Pharmacy News** (Tessa Hoffman). *Most pharmacists in breach of new CPD rule*. (10 comments) 22 August 2016. Accessed on 23 December 2016 at http://www.pharmacynews.com.au/news/latest-news/most-pharmacists-in-breach-of-new-cpd-rule.

Probably because it is a useless process that has been poorly outlined and explained by the Pharmacy Board. PSA has a CPD planning tool which is laborious and unhelpful. Yet they tell us if we don’t use it, we’re probably doing it wrong.

Surely we all do CPD that is relevant to our practice, or to practice that we’re interested in moving into. Why would we waste our time doing anything else?

We have no time and no motivation for CPD plans, especially since it is all for a pay-packet that we could get at the local supermarket.

For soooo many to not understand means it hasn’t been explained very well at all!!!!

The Guild’s MYCPD is still developing a tool so that’s part of my reasoning of not having a plan as yet .... not going to do the process twice, don’t have the time and don’t get paid for it .... hard enough to make a buck as it is!!

That’s because it is nonsense. I refuse to write a plan and will continue to study and read what takes my fancy or I feel needs refreshing. It’s [a] fluid thing—i.e., meet [a] patient with a certain condition and research it.

Attend a conference that takes my fancy. Do a post graduate uni course that presents itself. It’s not planned but adjusted to need in an environment of continual learning. Whoever comes up with this crap needs a life and to stop finding more crap for us to do instead of helping patients.

I mean who seriously has the time to sit down and plan their CPD footprint for the next 12 months. Get a life people. I’ll take the fine any day. It’s not like my $25/hr job is that great anyway. Worse comes to worse I’ll just get a job at Bing Lee.

Seems that our regulators have run out of good and worthwhile things to do—and run out of people to annoy all at the same time. Someone or some committee must have needed to justify their ongoing employment. Thus a further load of regulatory bs has been added to that which already exists.

Having worked through the PSA “tool” and having developed a “plan” I am in the compliant 10% and am happy to have given assorted regulators and software “tool” developers something to do for the time being but I wonder what new schemes are being dreamt up to bother us next year and in years to come

That approx. 90% appear not to be compliant tends to demonstrate that the CPD plan and the PGA and PSA tools are bad policy and not acceptable to the majority and that the whole “plan” concept needs to be re-evaluated for real life relevance

Now I assume that the regulators and software developers are actually being paid—unlike the rest of us who must do CPD in our own unpaid time and pay to attend courses etc.—so there’s probably little realisation of the financial and social costs of CPD schemes as they apply to the profession as a whole—maybe there needs to be a cost/benefit study into CPD before any more regulatory schemes

When we develop an in-store promotion or a protocol to achieve a desired outcome we plan the event. We start with a need, weed out the non-essential elements and hone the process until we have a desired workable system. The CPD plan is nothing but an extension of that process. It is to ensure we develop in the best path possible so that we can deliver our knowledge, remain focused and enthused in our profession and not wander off on unproductive tangents. The only difficulty at the moment is the means to develop this, just as professional bodies are also still formulating, but we should be able to jots down personal guideline and ideas as we wait patiently for a definitive checklist or table. Good luck with yours. I realise how challenging it is as I believe that even though I have one, I need to continually monitor and work on it as situations and demands on my talents change.

I think a bigger concern is the current expectation for plan + CPD exceeds all other areas of allied health ... whilst getting paid less.

Anyone who has any clinical responsibility where there recommendation are held to account by medical teams does this automatically. It is not challenging it is a waste of time.

Actually I need sit down and partake in dribble. I learn, and study and do plenty of CPD but I don’t waste time writing a plan. I don’t feel lacking in any areas to plan. Things present themselves and it is only then you realise you don’t know enough so you research it. If you see an interesting conference you do it, as CPD pops up on various websites you follow what takes your fancy. A course comes up that is relevant you do it, I don’t waste time planning and documenting and fluffing about, I get on with it. I don’t waste time recording interventions either as we do to many being in a medical centre and time is precious.

Over documentation and fakery are the nonsense bull dust of the modern world. Wasting time, money and intentions.

Some people might operate that way and it works for them but for me I feel like screaming every time a new rule is introduced to complicate life.

No time for this kind of craps. I have my own plan in my head. I don’t need to write it down as proof to show the board. Pharmacists are not kids and the Board is not our parents. I don’t waste my time that I can use to treat my patients. At home, I need time for my family. Otherwise, my wife would divorce me. Believe it or not, it is true. Will the Board pay me the divorce fee? If not, then leave all pharmacists alone. With only $25 an hour, don’t ask too much from pharmacists. Otherwise, everyone will quit and work for Aldi or BingLee. Same money, but less responsibilities and less crap.

**Pharmacy News** (Tessa Hoffman) *Six things to know about your CPD plan*. (22 Comments) 23/08/2016. Accessed on 23/12/2016 at http://www.pharmacynews.com.au/news/latest-news/six-things-to-know-about-your-cpd-plan.

When did Intern supervising get taken off? If that’s the case, I have a lot of hours to do in the next month.

The template is headed ‘Plan/Record’. Are we required to make a record of the past year or a plan for the next year?

Seriously, could there be any more work they want to load on us for the measly $27/hr???!!! I can get that wage at Aldi without all this headache.

Pharmacy is a dead end job!!! Get out while you can.

I really find this ridiculous—I refuse to write a plan. Do they not realise people will write what ever and just dribble in then go about what they do normally anyway? Just like QCPP? All words and fluff and no substance.

I do so much CPD more than I do work actually and spend considerable money on it. I refuse to write a plan. It’s ridiculous. I can now either lie like most people will or send a copy of my new postgraduate degree and say this should bloody cover it. It makes my blood boil. I think everyone should refuse. They can’t deregister us all

Absolutely agree. I doubt that anyone who actually works full time in the profession was involved in the development of this rubbish.

No, I don’t think I’ll be doing this. Undertake and record my mandated yearly points, that’s what I’ll do.

How do you decide what CPD to do? Is it anything that is timely and convenient, or to fill gaps in your knowledge and skills?

Thanks to HMR caps, expensive CPD events/post grad courses were the first thing I culled despite their clinical relevance and profession expansion. Clinical knowledge and practice is not monetarily rewarded, nor is it recognised by the PGA, AACP, PSA in terms of career expansion .... so why put any time and money into it?

Knowledge is power. And that can bring rewards. Think for example about the pilot sites for health care homes announced yesterday. How will practices choose a pharmacist to be part of the HC home? It will be based on your reputation and capacity to contribute to the team. They will be looking for pharmacists with a high level of clinical knowledge.

No, it will be decided as all these things are by informal networks between Pharmacy owners and GP practices. Employee Clinical Pharmacist input = Zero

Knowledge is power, at least in most industries. Absolutely reputation will help you gain a position such as this. However, I’m not holding my breath regarding the HC homes until I see the remuneration offered. Only then can we gauge how the government values clinical pharmacist input. With HMRs being one of the most valuable services available...culling them to me sends a pretty clear message. This move was met with very little resistance from all pharmacy bodies (including accreditation bodies) which sends an even bigger message. This would not have happened in any other industry. So it would appear clinical knowledge gives you very little power where it matters most.

On a side note anyone know the role of the AACP? They didn’t release a position statement when the caps were announced. Their fees haven’t decreased since the caps. All I can see is that they release an occasional newsletter and email regarding upcoming expensive CPD programs they are holding to receive more money (I’m yet to see a medical consultant on the list of speakers, who often offer far greater insight into medication use in their field than a pharmacist).

The only thing Pharmacy owners ever ask prospective Pharmacist employees is how many scripts can you dispense per hour. No one cares about your clinical skills or knowledge nor is it reflected in the Pharmacy award which has become not only the legal minimum but the de facto pay rate for many.

I never, ever, ever do the business-related CPD. It has SFA to do with my practice as a pharmacist and to be frank is insulting that it is scored the same in terms of development in learning about a new drug class, treatment regimen, or health topic.

If I come across a topic I feel I don’t know enough about—that’s my CPD. Hard to plan and map that, because like Uncle Don said “There’s things you know you don’t know, and there’s things you don’t know you don’t know”.

That is what people will do regardless—they will just pretend to plan it at the end of the year. I also feel you don’t know what you want to learn until you realise you don’t know it—then you immediately rectify the situation. Such as coming across a drug or a condition you need to know about but don’t. You don’t plan to study it later in the year to open google and get to it. You target your CPD to your interests and practice anyway.

I understand some people might like to operate that way but for me, useless meaningless paperwork I will not partake of.

Those that are not keeping will not change their practice either—They will just lie.

I do CPD based on (admittedly, self-ID’d) knowledge gaps. Personal and family commitments mean that for me a plan is too constricting (ie. do x at time y), and sometimes things that I thought I needed to study two months ago don’t actually require the active study I thought they did because of the passive intake of information (like Zika for instance—bits and pieces over months complete the picture, rather than a block of time to swot the subject). I’m taking a calculated risk and if I’m audited that’s fine, that’s the game.

**Pharmacy News** (Tessa Hoffman) *Pharmacists Lash Out* (16 Comments), 24/8/2016. Accessed on 23 December 2016 at; http://www.pharmacynews.com.au/news/latest-news/pharmacists-lash-out-against-new-cpd-rule.

All it will do is make people consider what the most relevant training is for them .... and then make them reconcile that against competency standards. And then make them document what they did. And then make them document how it changed their practice. No, that won’t be time consuming at all ....

Why can’t pharmacy just go back to voluntary CPD? Instead of this “Big Brother with the Big (deregistration) Stick” approach.

Umm, I just made my plan; “Get to 40 CPD points” Isn’t that sufficient?

I think this the ultimate “nanny” phenomena. If we accrue 40 points, subject finished requirements met. If you can’t meet the 40 points without a plan you shouldn’t be a registered pharmacist “GO HARD OR GO HOME”.

Anyone with any level of clinical accountability who is not actively seeking to fill knowledge gaps and expand should not be practicing. Direct HMR referrals uncapped would assist with this over “CPD plans” ... call itself auditing by GPs. Written plans are useless. Most will be completed in hindsight based on what was completed earlier in the year.

As pharmacy continues to get raped this plan is ridiculous! CPD is expensive and time consuming as it is. Accredited pharmacists need to complete 60 CPD points .... can only see 20 patients a month and now require a plan! What a joke!!!!

Here’s perspective physio CPD is 20 hours a year

Since we are scientists and deal with evidence-based therapies, can AHPRA or the Pharmacy Board provide us with studies that prove that pharmacists with a CPD plan perform better or are more up to date than those who don’t have a CPD plan?

Time and time again we see examples of how some moron trying to justify their salary and position on the board come up with some stupid idea that gets supported by other idiots who think they are actually more important than they really are. The new requirement for a compulsory CPD plan is yet another example.

I’ve yet to meet one pharmacist who believes in the idea who doesn’t have some other agenda they’re working on (e.g., seeking a position on the board or some academic position because they themselves can’t stand working in community pharmacy anymore).

All the negative comments from other readers to date will as usual be ignored. The point that our pay is a joke is always rebutted with the argument that we should be happy to be working at all or that we are lucky to be in a respected job. Well I’ve got news for you—pharmacy is definitely not what it used to be. The only people I feel worse for are the pharmacy assistants who work their backsides off for the lowest pay rates in the country.

We are all expected to perform a lot more for a lot less these days and sell out our dignity in the process (e.g., Generic substation/“value plus”, discount pharmacy models, etc.). This is apparently the better working standard that is the trade-off for the low pay we’re expected to be content with.

Back to the compulsory CPD plan debate—it won’t achieve anything other than waste pharmacists’ time. The idiot who drafted it will somehow put a spin on it with fancy stats to indicate how it will improve pharmacy practice but it will be a lie. Every pharmacist I’ve spoken to has indicated that if push comes to shove and they have to do it the only way they can draw up a plan is in retrospect i.e., do the compulsory CPD during the year to get the points then work backwards to come up with a plan. How else could it work if you don’t even know what CPD activities are available at the start of the year? So if most pharmacists are planning to come up with the plan in this way how does it achieve anything other than waste all our valuable time. And while on the topic of time could someone please work out what our actual hourly rates are after factoring in all the hours we spend on compulsory CPD as well as the money spent to attend activities, pay for annual registration, professional indemnity insurance, and so on and so on.

Pharmacy is the joke among all university studied professions!

Year after year I see disillusioned young men and women come out into the pharmacy workforce regretting their choice of career.

For myself I only work in pharmacy on weekends because the penalty rates (while they still exist) at least make it bearable. I have a second job in another industry during the week so that I can maintain a half decent lifestyle otherwise I would fall below the poverty line like all our poor pharmacy assistants.

I’m a casual locum ......... I’m stuffed if I can satisfy the Boards multi-level requirements in their “PLAN” ....... I have had no problem obtaining 100 plus Points annually since CPD started, you can’t tell me I’m not trying!!

CPD’s should only be used as a punishment system for pharmacists who have demonstrated negligent practices. Everyone else has demonstrated that the knowledge they acquired studying to become b a pharmacist or due to their experience as being a pharmacist is sufficient enough for the health care needs of their workplace and demographic.

I do believe we need to keep up-to-date and do a lot of CPD to [keep] up to speed and because I like to learn but these plans are just bull shittery concocted up by some numpty with ocd.

The board is dreaming if they think their $26 pharmacists will spend a single minute on this rubbish. Hell being deregistered could be a blessing in disguise. You’ll probably earn more elsewhere anyways.

No time for this kind of crap. I have my own plan in my head. I don’t need to write it down as proof to show the board. Pharmacists are not kids and the Board is not our parents. I don’t waste my time that I can use to treat my patients. At home, I need time for my family. Otherwise, my wife would divorce me. Will the Board pay me the divorce fee? If not, then leave all pharmacists alone. With only $25 an hour, don’t ask too much from pharmacists. Otherwise, everyone will quit and work for Aldi or BingLee. Same money, but less responsibilities and less craps.

If the board has time to audit pharmacists regarding the CPD plan, I am wondering why they don’t audit Chemist Warehouse for compliance of Pharmacy Standards? Pharmacists in Chemist Warehouse don’t counsel patients at all. It is not their fault, but the fault of the founders of Chemist Warehouse.

Pharmacists with no CPD plan do not cause any harm to patients, but Chemist Warehouse giving out medications without counseling would cause harm.

I myself don’t have time to write down the CPD plan. I won’t do it until the Board provide proof that all other pharmacists have the plan. I have the same plan as “Ain’t nobody got time for dat” in this forum, namely: Get to 40 CPD points. Simple!!!

**AJP.com.au** (Sheshtyn Paola) *Pharmacy Board responds to CPD plan uproar* (7 Comments), 29/08/2016. Accessed on 23/12/2016 at https://ajp.com.au/news/pharmacy-board-responds-cpd-plan-uproar/.

Can I point out that a plan is personal to you and your circumstances. It can be simple or complicated and you sure don’t need a workshop to make one. I’m pretty sure you are not going to be marked on it! Bit of common sense needed in this discussion.

Exactly, it’s mindless busy work.

The revised registration standards and CPD guidelines followed a rigorous public consultation process

I agree that we were informed early enough of the need to have a CPD plan, however I strongly dispute the suggestion that this followed a “rigorous consultation process”. Who exactly was consulted. I certainly wasn’t, nor was I aware that there was a consultation process.

Bureaucratic time wasting. Next they’ll have us write a protocol for attending CPD events, requiring a plan on where we sit, when we started sitting and how it impacted our learning by sitting in that spot.

SHPA also has a package of material to support members prepare a learning plan—including an online presentation that explains in detail how to develop a learning plan and competency grids for a range of pharmacist roles.

## References

[1] Nash R., Chalmers L., Brown N., Jackson S., Peterson G. (2015). An international review of program wide use of competency standards in pharmacy education. Pharm. Educ. J..

[2] (2010). National Competency Standards Framework for Pharmacists in Australia. http://www.psa.org.au/supporting-practice/national-competency-standards.

[3] Pharmaceutical Society of Australia (2010). Professional Practice Standards. https://www.psa.org.au/supporting-practice/professional-practice-standards.

[4] Australian Commission on Safety and Quality in Health Care (2012). National Safety and Quality Health Service Standards. https://www.safetyandquality.gov.au/our-work/accreditation-and-the-nsqhs-standards/resources-to-implement-the-nsqhs-standards/#NSQHS-Standards.

[5] Ryan J. (2003). Continuous professional development along the continuum of lifelong learning. Nurse Educ. Today.

[6] Rouse M.J. (2004). Continuing professional development in pharmacy. Am. J. Health Syst. Pharm..

[7] Driesen A., Verbeke K., Simoens S., Laekeman G. (2007). International Trends in Lifelong Learning for Pharmacists. Am. J. Pharm. Educ..

[8] Meštrović A., Rouse M.J. (2015). Pillars and Foundations of Quality for Continuing Education in Pharmacy. Am. J. Pharm. Educ..

[9] Konstantinides G. (2010). Continuing Professional Development: The role of a regulatory board in promoting lifelong learning. Innov. Pharm..

[10] Institute of Medicine (2009). Redesigning Continuing Education in the Healthcare Professions. http://www.policymed.com/2009/12/institute-of-medicine-redesigning-continuing-education-in-the-health-professions.html.

[11] Siemens G. Connectivism: A Learning Theory for the Digital Age. http://er.dut.ac.za/bitstream/handle/123456789/69/Siemens_2005_Connectivism_A_learning_theory_for_the_digital_age.pdf?sequence=1&isAllowed=y.

[12] Wiedenmayer K., Summers R.S., Mackie C.A., Gous A.G., Everard M., Tromp D. (2006). Developing Pharmacy Practice: A Focus on Patient Care: Handbook.

[13] Pharmacy Board of Australia (2015). Guidelines on Continuing Professional Development. http://www.pharmacyboard.gov.au/News/2015-10-30-registration-standards.aspx.

[14] Kolb D.A., Kolb D.A. (1984). Experiental Learning: Experience as the Source of Learning and Development.

[15] Pharmacy Board of Australia Continuing Professional Development (CPD) FAQ. http://www.pharmacyboard.gov.au/Codes-Guidelines/FAQ/CPD-FAQ.aspx.

[16] Biggs J., Tang C. (2011). Teaching for Quality Learning at University: What the Student Does.

[17] Harden R., Crosby J., Davis M. (1999). AMEE guide No. 14: Outcome-based education: Part 1—An introduction to outcome education. Med. Teach..

[18] Australian Government Department of Education and Training (2015). Higher Education Standards Framework (Threshold Standards).

[19] Australian Pharmacy Council Accreditation Standards for Pharmacy Programs in Australia and New Zealand. http://pharmacycouncil.org.au/content/index.php?id=17.

[20] Jackson D. (2013). The Contribution of Work-Integrated Learning to Undergraduate Employability Skill Outcomes.

[21] Freudenberg B., Brimble M., Cameron C., MacDonald K.L., English D.M. (2013). I am what I am: Am I? The development of self-efficacy through work integrated learning. Int. J. Pedagog. Curric. (Sect. Int. J. Learn.).

[22] Wilkens S., Tucci J. (2014). Group Project—Learning Research and Generic Skills for Life beyond University. Pharmacy.

[23] Kehoe A., Goudzwaard M. (2015). ePortfolios, Badges, and the Whole Digital Self: How Evidence-Based Learning Pedagogies and Technologies Can Support Integrative Learning and Identity Development. Theory Pract..

[24] Chen H., Grocott L., Kehoe A. (2016). Changing Records of Learning Through Innovations in Pedagogy and Technology. Educause Review. http://er.educause.edu/articles/2016/3/changing-records-of-learning-through-innovations-in-pedagogy-and-technology.

[25] Oliver B., von Konsky B.R., Jones S., Ferns S., Tucker B. (2009). Curtin’s iPortfolio: Facilitating student achievement of graduate attributes within and beyond the formal curriculum. Learn. Communities Int. J. Learn. Soc. Contexts.

[26] Scott G. (2015). Powerful Assessment in Higher Education: Identification of Case Studies of Productive Approaches within and Beyond Australia.

[27] Fernandez N., Dory V., Ste-Marie L.G., Chaput M., Charlin B., Boucher A. (2012). Varying conceptions of competence: An analysis of how health sciences educators define competence. Med. Educ..

[28] Creswell J. (2013). Research Design Qualitative, Quantitative and Mixed Methods Approaches.

[29] Denscombe M. (2010). The Good Research Guide; Surveys and Sampling.

[30] Liamputtong P. (2013). Research Methods in Health: Foundations for Evidence-Based Practice.

[31] Nash R.E., Chalmers L., Stupans I., Brown N. (2016). Knowledge, use and perceived relevance of a profession’s Competency Standards; implications for Pharmacy Education. Int. J. Pharm. Pract..

[32] Braun V., Clarke V. (2006). Using thematic analysis in psychology. Qual. Res. Psychol..

[33] Australian Health Practitioner Regulation Agency The Australian Health Practitioner Regulation Agency and the National Boards, Reporting on the National Registration and Accreditation Scheme: Annual Report 2010–2011. http://www.ahpra.gov.au/Publications/Corporate-publications/Annual-reports.aspx.

[34] Thompson W., Nissen L., Hayward K. (2013). Australian pharmacists’ understanding of their continuing professional development obligations. Aust. J. Pharm..

[35] Australian Qualifications Framework Council (2013). Australian Qualifications Framework Second Edition I.

[36] Hattie J. (2008). Visible Learning: A Synthesis of Over 800 Meta-Analyses Relating to Achievement.

[37] Nash R., Stupans I., Chalmers L., Brown N. (2016). Traffic Light Report provides a new technique for Assurance of Learning. J. Learn. Des..

[38] Donyai P., Herbert R.Z., Denicolo P.M., Alexander A.M. (2011). British pharmacy professionals’ beliefs and participation in continuing professional development: A review of the literature. Int. J. Pharm. Pract..

[39] Collins J. (2009). Lifelong Learning in the 21st Century and Beyond. RadioGraphics.

[40] Bembenutty H. (2011). Introduction: Self-regulation of learning in postsecondary education. New Direct. Teach. Learn..

[41] Boud D., Falchikov N. (2006). Aligning assessment with long-term learning. Assess. Eval. High. Educ..

[42] Dopp A.L., Moulton J.R., Rouse M.J., Trewet C.B. (2010). A five-state continuing professional development pilot program for practicing pharmacists. Am. J. Pharm. Educ..

[43] Hughes C., Barrie S. (2010). Influences on the assessment of graduate attributes in higher education. Assess. Eval. High. Educ..

[44] Austin Z., Gregory P. (2007). Evaluating the Accuracy of Pharmacy Students’ Self-Assessment Skills. Am. J. Pharm. Educ..

[45] Laaksonen R., Bates I., Duggan C. (2007). Training, clinical medication review performance and self-assessed competence: Investigating influences. Pharm. Educ..

[46] Pfleger D., McHattie L., Diack H., McCaig D., Stewart D. (2008). Views, attitudes and self-assessed training needs of Scottish community pharmacists to public health practice and competence. Pharm. World Sci..

[47] Schack D., Hepler C. (1979). Modification of Hall’s Professionalism Scale for Use with Pharmacists. Am. J. Pharm. Educ..

